# Radioresistance and brain metastases: a review of the literature and applied perspective

**DOI:** 10.3389/fonc.2024.1477448

**Published:** 2024-10-30

**Authors:** Andrew Youssef, Arjun Sahgal, Sunit Das

**Affiliations:** ^1^ Institute of Medical Science, University of Toronto, Toronto, ON, Canada; ^2^ Department of Radiation Oncology, Odette Cancer Centre, Sunnybrook Hospital, Toronto, ON, Canada; ^3^ Division of Neurosurgery, St. Michael’s Hospital, University of Toronto, Toronto, ON, Canada

**Keywords:** radiotherapy, cancer, brain, metastases, radioresistance

## Abstract

Intracranial metastatic disease is a serious complication of cancer, treated through surgery, radiation, and targeted therapies. The central role of radiation therapy makes understanding the radioresistance of metastases *a priori* a key interest for prognostication and therapeutic development. Although historically defined clinic-radiographically according to tumour response, developments in new techniques for delivering radiation treatment and understanding of radioprotective mechanisms led to a need to revisit the definition of radioresistance in the modern era. Factors influencing radioresistance include tumour-related factors (hypoxia, cancer stem cells, tumour kinetics, tumour microenvironment, metabolic alterations, tumour heterogeneity DNA damage repair, non-coding RNA, exosomes, methylomes, and autophagy), host-related factors (volume effect & dose-limiting non-cancerous tissue, pathophysiology, and exosomes), technical factors, and probabilistic factors (cell cycle and random gravity of DNA damage). Influences on radioresistance are introduced and discussed in the context of brain metastases.

## 1 Introduction

### 1.1 Intracranial metastatic disease and radiotherapy

Intracranial metastatic disease (IMD) is a serious complication of cancer and the most common type of brain tumour (1), estimated to affect roughly 2% of all patients with cancer and compromise 12% of all metastatic sites (2). IMD has a poor prognosis, which is affected by factors such as the extent of systemic disease, number of intracranial metastases, performance status and age (1, 3). Treatments for IMD include surgery, radiation and target therapies (4). Radiation therapy (RT) holds a central role in IMD treatment and why understanding the radioresistance of a metastases *a priori* is of key interest in both prognostication and therapeutic development.

### 1.2 History of radiobiology and radioresistance

The effects seen from RT result principally from direct or indirect DNA damage (5). Direct ionising radiation damage results from DNA ionisation or excitation, leading to a base change. Indirect ionising radiation damage occurs more frequently from RT and occurs when the energy from the RT is transferred to other cellular atoms (namely water) and produces free radicals, which can then damage the DNA (5).

Historically, radioresistance has been defined clinic-radiographically according to tumour response following treatment with a maximum tolerable dose (6). However the radiation dose was limited by conventional techniques. In particular for brain metastases the treatment was whole brain radiation treatment (WBRT), and the “maximum” dose applied was far lower than what can be achieved with modern conformal stereotactic techniques. Therefore, the development and increasing use of stereotactic radiosurgery (SRS), which allows a higher maximum tolerable dose because of decreased radiation exposure beyond the target, has brought into question the validity of these classical definitions. There is a need to revisit the definition of radioresistance in the modern era.

**Figure 1:**
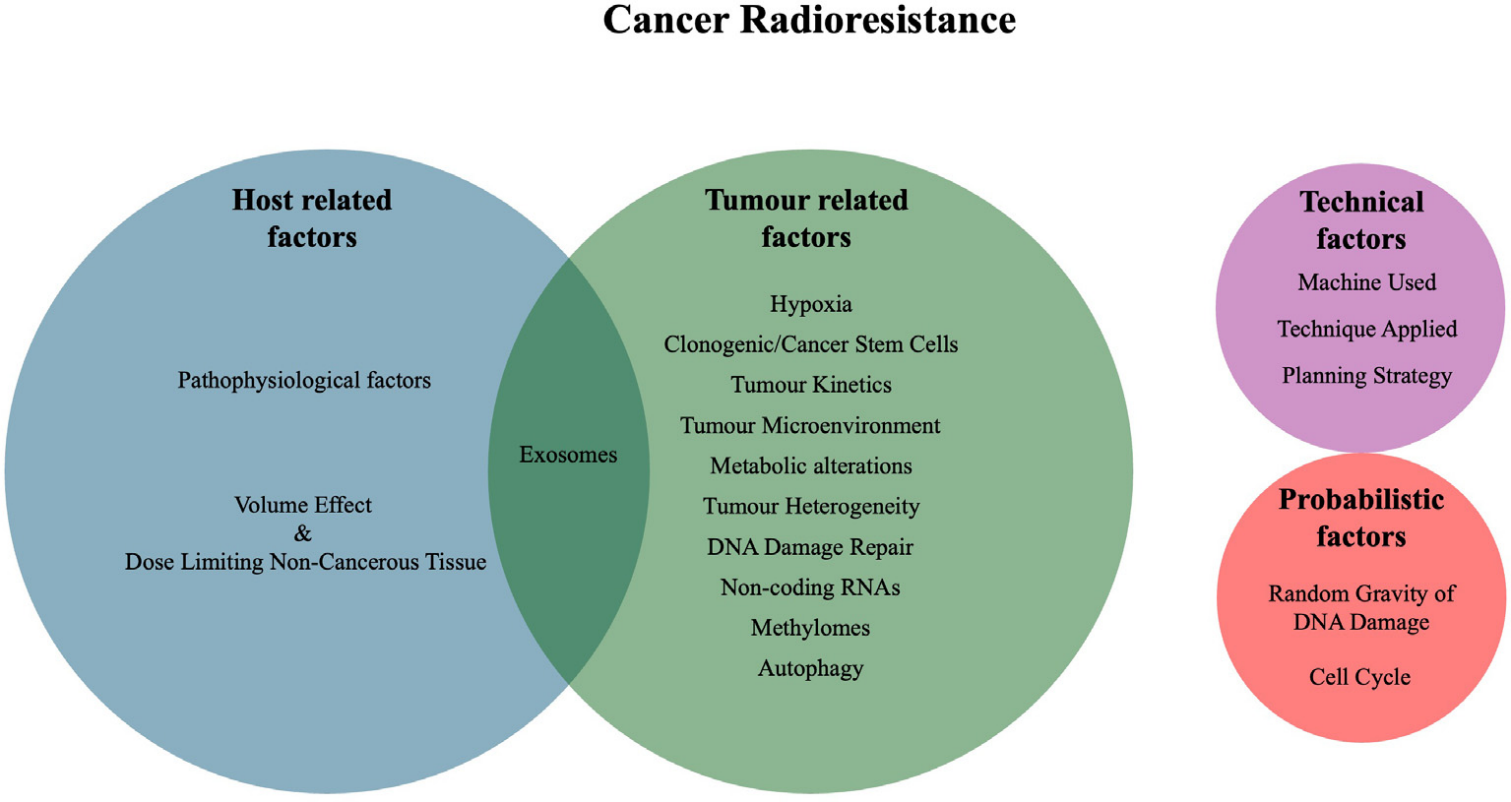
Venn diagram of the mechanisms and factors of cancer radioresistance.

Lester J. Peters, in his keynote address from the 1980s, identified four main categories of clinical radioresistance: tumour-related factors, host-related factors, technical factors, and probabilistic factors (6). Tumour-related factors include hypoxia, number of clonogenic cells, tumour kinetics and intrinsic radioresistance. Host-related factors include the volume effect (complications based on the volume irritated), dose-limiting non-cancerous tissue, pathophysiological factors and host defences. Technical factors include geographic miss of treatment and errors in dose delivery. Lastly, probabilistic radioresistance, as RT relies on damaged DNA to produce an effect, speaks to the randomness inherent in the production of DNA damage (6). Since then, additional factors that affect radioresistance have been identified, including the tumour microenvironment (TME), metabolic alterations, cancer stem cells (CSCs), tumour heterogeneity, microRNAs, cell cycle stage, DNA damage repair pathways, exosomes, methylation state and autophagy (7–9). Ultimately, many of these factors are interconnected and influence each other through various mechanisms and sub-pathways (**Figure 1**).

## 2 Tumour-related factors

Peters identified hypoxia, the number of clonogenic cells, tumour kinetics and intrinsic radioresistance as the tumour-related factors causing radioresistance. Work performed since has further characterised the mechanisms in which tumours develop radioresistant phenotypes (6). The broad term of intrinsic radioresistance, which had initially been defined as “radioresistance manifested by asynchronous well-oxygenated populations of tumour cells cultured *in-vitro*” and had focused on DNA repair mechanisms (6), now also includes factors such as the TME, metabolic alterations, cancer stem cells (which had been previously termed clonogenic cancer cells), tumour heterogeneity, DNA damage repair, non-coding RNAs, exosomes, methylation state and autophagy.

### 2.1 Hypoxia

Indirect DNA damage inflicted by RT is primarily mediated by reactive oxygen species (ROSs), including O^2-^, H_2_O_2_, and OH (8). The ionising effect of RT is enhanced by normoxia (the “oxygen effect”) (5). The “oxygen fixation hypothesis” asserts that DNA damage incurred by free radicals is made irreparable when free molecular oxygen is available (5, 10). In many brain metastases, however, the TME is marked by regions of relative hypoxia (11). Hypoxia in tumours occurs in a gradient in relation to capillary penetration, leading to levels of cellular oxygenation ranging from normoxia to anoxic necrosis (5, 12). This gradient can result in varied responses to RT (5). Acute cellular hypoxia may occur regardless of cell proximity to the vasculature, adding a layer of temporal hypoxia that may alter treatment response (13).

Further, hypoxia may induce many pro-proliferative or adaptive pathways that can enhance radioresistance. Activation of hypoxia-inducible factor-1 (HIF-1), for example, may result in multiple cell-level changes, such as in energy metabolism, cell cycle, epithelial-to-mesenchymal transition (EMT), DNA damage response, autophagy, epigenetics and cytoprotection (11), that can support cell survival in hostile microenvironments, all while aiding in the maintenance of a stem-like state (14). Chronic hypoxia and vascular remodelling can also cause endothelial and oligodendrocytic cell death, resulting in demyelination and defective microenvironment function (15). In studies evaluating hypoxia in lung cancer-derived brain metastases, hypoxia was found to be a considerable yet heterogeneous feature.

### 2.2 Clonogenic/Cancer stem cells

Clonogenic cells are defined as cells that are capable of regenerating the tumour within which they are found (6). More recently, this property has been proposed to reside within cancer stem cells (CSCs). CSCs have also been shown to possess intrinsic resistance to cytotoxic injury and cell death (16) and, as such, may serve as a reservoir for tumour recurrence following RT (17). CSCs employ a variety of radioresistance mechanisms to survive radiation-associated injury (17). Relative to non-CSCs, CSCs have an enhanced capacity for DNA repair and defences against ROS-mediated DNA damage (18).

CSCs have been identified to express many molecular mediators of radioresistance, including components of the ERK, VEGF, glycolysis, WNT/β-catenin, Notch, JAK2 and PI3K/Akt/mTOR pathways (19, 20). Markers for CSC radioresistance include CD133, CD24^-/low^/CD44^+^, CD29, CD44, CD45, CD3, CD20, CD10, glycophorin A, CD64, CD 326, and ALDH1 (19). CSCs have also been found to be capable of undergoing the epithelial-mesenchymal transition (EMT), which has been linked to the development of metastatic disease (14).

### 2.3 Tumour kinetics

Due to radiation treatments being delivered through fractions over a timeframe, tumour cells are given opportunities to replicate between treatments (6). The more active the tumour kinetic profile (i.e. growth rate), the more ‘recuperation’ can occur between treatments (21). Relative to fractionated WBRT, SRS delivers a high radiation dose in a single treatment (or a few high dose per fraction treatments). Intracranial progression in those with IMD is common in the timeframe between diagnosis and the day of SRS treatment, with an interval growth in the index lesion (largest lesion at diagnosis) of a minimum of 3mm in roughly 60% of the patients (22).

Tumour kinetics of IMD can vary based on the primary tumour type. A study investigating apparent diffusion coefficient (ADC) in brain metastases pre- and post-SRS, found that ADC was higher pre- and post-SRS in brain metastases relative to healthy brain tissue, while no significant difference in ADC pre-SRS was observed between samples of different primaries. Interestingly, post-SRS, lung metastases ADC decreased, breast metastases ADC increased, and genitourinary metastases ADC did not significantly change (23). Differences in IMD kinetics have been found to be a useful prognostic indicator for overall survival in patients receiving SRS for IMD, with a study finding the hazard ratio per 1% change in brain metastases size/day between diagnostic and stereotactic MRI to be 1.32 (95% CI 1.06-1.65) (24).

### 2.4 Tumour microenvironment

The TME plays a role in tumour phenotype and overall cancer outcomes while modulating factors such as hypoxia and influencing the behaviour of tumour cells (13). Influences can come from cross-talking cancer cells, immune cells, vasculature, structural tissue, and more (13). Many TME components are mixed in with the cancer cells, with varying spatial architecture of the TME between lesions (25). Unbalanced cancer cell proliferation leads to unbalanced oxygen demands, which neo-vasculature attempts to respond to (13). Neo-vasculature is developed in response to proinflammatory signals such as vascular endothelial growth factor (VEGF), secreted by tumour cells (26). This vasculature provides oxygen and nutrients but rarely permeates the tumour, meaning the aforementioned hypoxic gradient remains in the presence of neo-vasculature (13).

Tumour-associated macrophages (TAMs) have been well characterised for their prevalence and association with cancers, their ability to promote tumour growth, and their role in enhancing radioresistance (13). Cyclooxygenase-2 (Cox-2) can be produced in cells such as macrophages, producing prostaglandin E_2_ (PGE_2_), which can lead to the upregulation of early growth response1 (Egr1) transcription factor and Id1 expression, increasing resistance to radiation-induced DNA damage (27). It has also been suggested that irradiation of macrophages induces high expression of tumour necrosis factor-α (TNF-α), promoting angiogenesis and cell survival (28).

Studies on Cancer-Associated Fibroblasts (CAFs) and RT have reported a poorer prognosis following RT when increased levels of CAFs are detected (13). Factors secreted by CAFs reported to induce radioresistance include epidermal growth factor (EGF), fibroblast growth factor 4 (FGF4), and insulin-like growth factor 2 (IGF2) (29). These factors confer survival and protection signals to elude radiation-induced cell death (29).

Targeting the post-irradiation inflammatory process could potentially aid in the complete elimination of cancer cells by reducing the long-term fibrotic presence (30). Post-irradiation upregulation of NF-KB is associated with the expression of inflammatory cytokines and enzymes within the TME, such as IL-β/6/8, Cox-2, and granulocyte-macrophage colony-stimulating factor (30).

The brain is a somewhat unique structure in the body, composed of distinct tissue types (e.g. astrocytes, neurons, microglia), leading to an equally unique extracellular matrix (ECM) and TME (31, 32). Studies have found a loss of PTEN expression in tumour cells when in the brain, determining that microRNA containing exosomes released by astrocytes lead to this PTEN suppression (33). PTEN suppression aids in metastatic growth (32). Additionally, a study investigating the cellular architecture of IMD by analysing stromal composition observed that IMD from different cancer types presented with similar metastatic niches. All cases observed were mainly located in the brain’s cortex; however, major differences in immune composition were observed (34).

### 2.5 Metabolic alterations

Metabolic changes within cancer are a well-classified staple of the disease. The Warburg effect, through the promotion of glycolysis and synthesis of NADPH through the pentose phosphate pathway, provides a source of electrons for antioxidant systems, ultimately decreasing cellular ROSs (8, 35). Other glucose metabolism-related factors associated with radioresistance include the GLUT1 membrane protein (36). This protein has a high affinity for glucose, allowing for higher transport rates under normal physiological conditions (37).

Lipid metabolism also plays a role in cellular radioresistance (38, 39). Increased fatty acid oxidation (FAO) reduces fatty acid accumulation following RT (39). FAO products such as FAO-derived acetyl-CoA also aid in radioresistance through pathways enhancing anti-phagocytosis in radioresistant cells (39). Apart from lipid and glucose metabolism, radiosensitisation of cancer cells was also observed in a study where glutamine metabolism was targeted by inhibition of GLS and MYC (40).

Cellular metabolism in the brain primarily uses glucose as the desired energy source (41). A study on breast cancer IMD found an increase in the expression of enzymes involved with glycolysis, oxidative phosphorylation, and the tricarboxylic acid cycle when oxygenated and increased activation of the redox-balancing pentose phosphate pathway and glutathione pathway (42). Additionally, in the absence of glucose, brain metastatic breast cancer cells increased oxidations of glutamine and branched amino acids and gluconeogenesis to allow for non-oxidative pentose pathway synthesis of purine (43). Another study on breast cancer IMD, had found that the fatty acid binding protein 7 (FABP7) supports cellular glycolytic phenotype and storage of lipid droplets. This regulator aids in IMD development and initial survival within the brain microenvironment (44).

### 2.6 Tumour heterogeneity

The problem of progenitor cancer cells remaining after treatment can become exacerbated when heterogeneous populations of cancer cells reside within the same tumour. tumour heterogeneity spans a broad spectrum, including differences in molecular, cellular, genetic, and spatial features of cells (45). In a study on murine glioma radioresistance, single-cell transcriptomic analysis identified distinct radioresistant and radiosensitive groups. Of the radioresistant groups, one was highly proliferative, the other was not (12). Studies have found that tumour heterogeneity relies on the number of CSCs and that cell-cell and cell-matrix interactions lead to the tumour heterogeneity needed for growth (46).

A study performing a transcriptomic analysis during a study on breast cancer metastasis to the brain and liver, was able to generate 50 intratumoural expression-based subclusters for overall transcriptomic analysis across all tumour cells (47), exemplifying the sheer quantity of intratumoural heterogeneity possible within IMD. IMDs often have clinically relevant genotypic and phenotypic changes that occur during the process of metastases, which can contribute to observed heterogeneity. Performing whole-exome sequencing analysis between matched primaries and brain metastases, using 83 samples from 11 different primaries, a study found 53% of cases contained potentially clinically relevant alterations detected in the brain metastases not present in the matched primary (48).

### 2.7 DNA damage repair

DNA damage repair serves as a main mechanism for radioresistance with mechanisms including non-homologous end joining (NHEJ), homologous recombination (HR), single strand annealing (SSA), mismatch repair (MMR), nucleotide excision repair (NER), and base excision repair (BER) (10, 17). Of these, only NHEJ and HR repair double-strand breaks, with HR being less error-prone by using the homologous sequence of the damaged DNA’s respective sister chromatid as a template (10). A key part of DNA damage response is sensing and responding to damage effectively. Sensors transduce the DNA damage signal and sometimes directly recruit DNA damage repair proteins to the site of damage (49).

Studies have found frequent overexpression of BARD1 and RAD51 in IMD from breast cancer relative to both matched primary tumours and unlinked systemic metastases. These genes function to repair double-strand breaks, ultimately protecting IMD from permanent ROS DNA damage (50). Another group investigating DNA damage response gene signatures in colorectal brain metastases found that IMD presented with deficiencies in HR and MMR relative to the colorectal cancer primaries (51). These two studies demonstrate differences in DNA damage repair between primary cancers and their IMD, which may ultimately influence radioresistance profiles between primaries and IMD.

### 2.8 Non-coding RNAs

Non-coding RNAs, such as microRNAs modulate gene expression following transcription (8). MicroRNAs do not directly confer radioresistance or radiosensitivity, but by modulating gene expression, some microRNAs influence radioresistance through their involvement with related signalling pathways (52).

Long non-coding RNAs (lncRNAs) have been found to play an important role in cancer radioresistance. LncRNAs play roles in transcription, chromatin remodelling, and biological behaviour within the cell (53, 54). A study investigating 23 lncRNAs in 11 cancer types found 13 lncRNAs downregulated and 3 upregulated related to radiosensitivity, and 11 lncRNAs upregulated related to radioresistance (55). Notable lncRNA targets or pathways included PSEN1, EGFR, p53, MAPK1, and ERK. An expanded list of significant non-coding RNA and their targets, including those identified by the aforementioned study, (55) are listed in **Table 1**.

**Table 1:** Radioresistance/radiosensitivity related non-coding RNA names, type, target, affected pathway, and study.

RNA name(s)	Type of RNA	Target(s) and/or affected pathway(s)	Study
miR-124	MicroRNA	CDK4	Deng et al. (59)
miR-221, miR-222	MicroRNA	DNA-PKcs	Li et al. (117)
miR-125a, miR-1, miR-150, miR-425	MicroRNA	P53 (miR-125a only), cell cycle checkpoint response	Moskwa et al. (57)
SP100-AS1	LncRNA	miR-622 & ATG3	Zhou et al. (118)
CircRNA 100367	Circular RNA	miR-217, Wnt3	Liu et al. (119)
miR-29a	MicroRNA	YY1, CDK6	Chuang et al. (120)
miR-222	MicroRNA	PTEN	Wu et al. (121)
miR-125b	MicroRNA	ICAM	Shiiba et al. (122)
NEAT1	LncRNA	miR-204, ZEB1	Lu et al. (123)
TPTEP1	LncRNA	miR-106a-5p, P38 MAPK	Tang et al. (124)
Linc00312	LncRNA	DNA-PKcs	Guo et al. (125)
Linc00114	LncRNA	miR-203, ERK/JNK	Han et al. (126)
HOTAIR	LncRNA	miRNA-93, ATG12	Liu et al. (127)
Linc00473, miR-374a-5p	lncRNA, MicroRNA	SPIN1	Chen et al. (128)
FAM201A	LncRNA	miR-370, EGFR	Liu et al. (129)
MALAT1, miR-1	LncRNA, MicroRNA	SLUG	Jin et al. (130)
LincRA1	LncRNA	H2Bub/USP44	Zheng et al. (131)
NKILA	LncRNA	NF-κB	Yang et al. (132)
Linc00958	LncRNA	miRNA-5095, RRM2	Zhao et al. (133)
CCAT2	LncRNA	miR-145, p53	Wang et al. (134)
miR-200c, Linc02582	MicroRNA, LncRNA	CHK1	Wang et al. (135)
Linc00473	LncRNA	Wnt/β-catenin	Han et al. (136)
PVT1	LncRNA	MiR-515-5p, PIK3CA	Han et al. (137)

Of the previously identified non-coding RNAs involved in response to radiation, few studies have identified their involvement in IMD. Circular RNA circBCBM1 modulates miR-125a and, through inhibiting miR-125a activity, promotes breast cancer metastasising to the brain (56). Interestingly, in a study on miRs, which induce radioresistance in glioblastomas, overexpression of miR-125a promoted radioresistance (57). A study comparing microRNA signatures in brain metastases compared to primary non-small cell lung cancer found notable upregulation of miR-124-3p and downregulation of miR-150-5p in brain metastases relative to the primary (58). miR-124 has been identified to promote radiosensitisation of glioma cells through targeting CDK4, and overexpression of miR150 induces radioresistance through cell-cycle checkpoint upregulation (57, 59).

### 2.9 Exosomes

Exosomes released by cells contain components such as mRNA, proteins, DNA fragments, lipids, and non-coding RNAs, playing roles in many cancer attributes, such as cell-cell communication, progression, interactions with the stroma and TME, and signalling pathway activation (7). While under stress, exosome qualities change, with studies that have found exosomes released from radiation-treated cancer cells enhanced the migration of cells which received them (60), and it has been indicated that exosomes aid in establishing pre-metastatic niches (61). Radiation-induced exosomes play a pivotal role in characterising cellular radioresistance (62). Studies on DNA damage and exosome secretion in cancer cells have found that p53 activation in response to DNA damage can increase exosome production and secretion (63, 64).

Exosomes maintain multiple roles within IMD, ranging from aiding in the initiation of metastasis by aiding in the destruction of tight junctions and modulating cell migration (65, 66) to being associated with intracranial failure following RT (67). A study on lung cancer patients with IMD receiving whole brain RT analysed circulating exosome integrins, where they found that levels of integrin β3 were associated with survival and local intracranial control (67).

### 2.10 Methylomes

Following RT, the methylomic landscape of cells can be altered, potentially promoting treatment efficacy or causing radioresistance (68). Differences have been found between the methylomes of radiosensitive and radioresistant cancers, as well as differences between the methylomes of intrinsic and adaptive radioresistance (68). When comparing the methylome of differentially responding non-small cell lung cancer, a study found hypermethylation was typically identified in the promotor regions of tumour suppressor genes and hypomethylation in specific genes; hypermethylation was found to be more prevalent in radioresistant cells (69).

Studies have identified and validated differentially methylated genes between radioresistant and radiosensitive non-small cell lung cancer phenotypes, including the cell survival and antioxidant-related genes SERPINB5, S100A6, CAT, and BNC1 (69). Studies in oral cancer have found additional radioresistant methylation markers with the hypermethylation of FHIT in radioresistant cells (70).

A study found that YTHDF3, which promotes breast cancer brain metastases through various pathways is dependent on m^6^A methylation (71). Furthermore, a study on DNA methylation of prostate cancer IMD, found aberrant methylation within the IMD to be associated with the activity of the PRC2 complex as well as their mutational background. Ultimately, their results suggested a specific reprogramming requirement within primary tumours to form IMD (72).

### 2.11 Autophagy

Acting as a cellular recycling mechanism allows autophagy to confer both radioresistance and chemoresistance. Ultimately, this cellular self-preservation mechanism allows cellular population regrowth following treatments and harsh conditions (9). For example, the glucose-related protein GRP94 has direct implications in promoting IMD through the activation of pro-survival autophagy pathways (73). These studies further identify autophagy as a cause of IMD and a target for treatment.

### 2.12 Related therapeutic approaches

**Figure 2:**
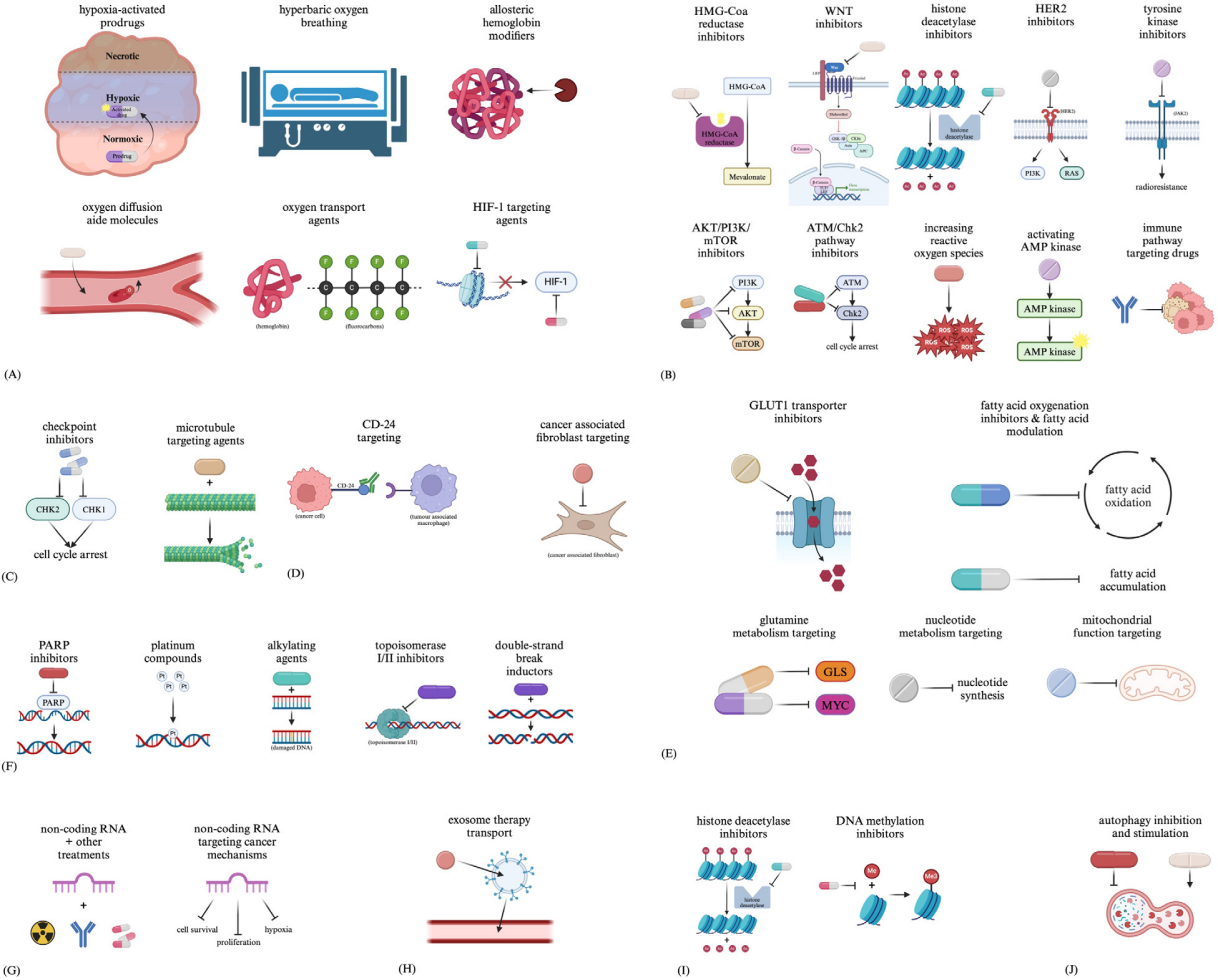
Therapeutic approaches related to **(A)** hypoxia, **(B)** CSCs, **(C)** the cell cycle, **(D)** the tumour microenvironment, **(E)** metabolic changes, **(F)** DNA damage repair, **(G)** non-coding RNAs, **(H)** exosomes, **(I)** methylation and **(J)** autophagy.

When targeting hypoxia, various approaches are being investigated, including hypoxia-activated prodrugs (HAPs), hyperbaric oxygen breathing, allosteric haemoglobin modifiers, oxygen diffusion aide molecules, and oxygen transport agents (74). With the importance of HIF-1 to hypoxia and overall tumour phenotype, it has also been identified as a target for drugs such as vorinostat and benzopyranyl triazole (75) (**Figure 2A**).

Targeted treatment of CSCs proves challenging due to the heterogeneity between subclones and how the tumour reacts to initial treatment (76). Although preclinical, papers review approaches under investigation for targeting CSCs in cancers such as acute myeloid leukaemia, including inhibition of HMG-Coa reductase, WNT, histone deacetylases, HER2, AKT/PI3K/mTOR, the ATM/Chk2 pathway; increasing reactive oxygen species, activating AMP kinase, and drugs targeting immune pathways (76). As tyrosine kinase pathways such as JAK2 have been implicated in CSC radioresistance, tyrosine kinase inhibitors may prove to be an additional radiosensitiser (**Figure 2B**).

Main targets for treatment aimed at the cell cycle include the cell cycle checkpoint kinases CHK1 and CHK2 (77). Additionally, many types of drugs can lead to cell cycle arrest, such as microtubule-targeting agents, indirectly altering the tumour kinetics of the affected cells (**Figure 2C**).

The TME is another target for therapies (78), including treatments such as immunotherapies with targets such as CD-24 to interfere with tumour cell and TAM interactions (79). Drugs in clinical trials targeting CAFs are also being developed, including small molecules aimed at preventing CAF activation, reprogramming CAFs, interfering with CAF-mediated signalling, and additional CAF-related functions (80) (**Figure 2D**).

Targets such as the GLUT1 transporter have been identified in many studies developing drugs to target this metabolic process (81–85). Aside from glycolysis-targeting agents, drugs aimed at targeting fatty acid synthesis and oxidation, glutamine metabolism, nucleotide metabolism, and mitochondrial function are all being investigated (81) (**Figure 2E**).

Drug types such as PARP inhibitors, platinum compounds, alkylating agents, topoisomerase I/II inhibitors, and double-strand break inductors have all been developed to target DNA repair. These drugs function by either indirectly targeting DNA damage repair by causing more DNA damage than can be repaired or directly targeting DNA damage repair by interfering with necessary mechanisms for repair (86) (**Figure 2F**).

Some studies have already begun researching targeting non-coding RNAs to act as sensitisers for other treatments (87). Additionally, non-coding RNAs have also been identified as potential therapies which could be used to target various cancerous processes (88) (**Figure 2G**).

Due to their ability to carry molecules between cells, strong tumour tropism, low toxicity and immunogenicity, and ability to travel throughout the body, exosomes are being investigated not as targets for treatments but as potential carriers for therapies such as biomolecules and chemical agents (89–91) (**Figure 2H**).

With the prominence of histone deacetylase (HDAC) inhibitors such as Vorinostat (92), along with DNA methylation inhibitors (93), modification of epigenetics and gene expression of cancer cells is also becoming a more prevalent route for novel cancer-treating drugs (**Figure 2I**).

Targeting treatment towards autophagy can prove difficult due to its opposing roles in both cancer progression and inhibition, leading to the proposal of interventions for inhibiting and stimulating autophagy (94). Drugs such as chloroquine and hydroxychloroquine are autophagy-inhibiting drugs (94), whereas stimulation of autophagy can generally occur through cellular stress (94), with molecules such as the semisynthetic vitamin E derivative alpha-tocopheryloxyacetic acid stimulating tumour autophagy (95). Additionally, a study reviewing the regulation of autophagy in intracranial tumours describes repurposing antidepressants with autophagy-modulating functions to induce cytotoxic effects through the dysregulation of autophagy pathways (96) (**Figure 2J**).

## 3 Host-related factors

Aside from the tumour’s inherent factors modulating response to radiation treatment, the indiscriminate damaging nature of RT leads to factors related to the host influencing the treatment parameters that can be used. This can be seen through factors such as the volume effect, dose-limiting non-cancerous tissue, and additional pathophysiological factors.

### 3.1 Volume effect and dose limiting non-cancerous tissue

The volume effect describes the increase in acute and chronic side effects of RT as treatment volume increases, namely due to larger amounts of non-cancerous tissue being irradiated (97). This problem can be manifested differently in different tissues, with various toxicities observed based on surrounding tissues (98). The significance of the brain and all of its functions makes minimisation and avoidance of neural toxicities an important consideration when planning neurological RT treatments.

Toxicities in the brain following RT can range from acute toxicities, such as fatigue, to more severe toxicities, such as cognitive impairment, necrosis of CNS tissue and disruption of endocrine function (98). Differences in precision and dosages between SRS and WBRT ultimately translate into different manifestations of toxicities. Techniques such as hippocampal-avoidance WBRT (HA-WBRT) have been developed to reduce cognitive impairment in patients receiving WBRT (99). The efficacy of HA-WBRT as a cognition-sparing approach has however, been questioned, with some studies finding no significant benefit to cognition relative to conventional WBRT (99). WBRT doses tend to range between 20 and 40 Gy, delivered in 5 to 20 fractions, with relatively lower precision (100), whereas SRS delivers high precision (accuracy of 1mm) and high radiation dosage in a single treatment (101).

In a study with patients with 1-3 IMD lesions, randomised to either SRS + WBRT or SRS alone to test differences in cognitive deterioration between the groups, SRS alone had less cognitive decline following 3 months relative to SRS + WBRT (102). In a separate study on long-term outcomes of patients with brain metastases randomised to either receive SRS or WBRT, less cognitive deterioration among long-term survivors was observed in those received SRS relative to those who received WBRT, with clinical meaningfulness with the association of late cognitive deterioration and WBRT (103).

Due to the different levels of precision and, ultimately, toxicities caused by these different treatments, dose limitations are placed to protect the surrounding tissue. Although doses being delivered are protective to surrounding tissue, if a tumour is presenting as radioresistant, these protective doses are likely insufficient to damage the tumour.

### 3.2 Pathophysiological factors

It has been well characterised how comorbidities can influence outcomes in cancer patients (104). Radioresistance of cancer is an additional outcome which can be influenced by comorbidities/pathophysiological factors such as anaemia and age (6). Examples include how patients with Fanconi Anemia tend to be relatively radiosensitive due to disruptions in DNA damage repair (105), and how elderly patients (both healthy and cancer patients) tend to be more radiosensitive in general (106).

### 3.3 Exosomes

Exosome transfer from stromal cells to breast cancer cells has been identified, with RNA within these exosomes modulated radioresistance within the breast cancer cells by regulating the STAT1/NOTCH3 pathway (107). Additionally, the microRNA miR-208a, which can be transported by exosomes, can modulate radiosensitivity and proliferation of lung cancer cells through targeting p21 and altering the AKT/mTOR pathway (108).

## 4 Technical factors

There are several considerations with respect to technical factors that can impact how we interpret outcomes. For example, doses can be prescribed in various ways, leading to homogeneous or inhomogeneous dose distributions, and there can be variation in the definition of the gross target volume (GTV), clinical target volume (CTV) and planning target volume (PTV). Even the technology used can impact biological effects on the tumour; for example, a study has found transcriptomic differences in SRS-treated oligo brain metastases between those receiving treatment through Gamma-knife (GK) and linear accelerator (LINAC) modalities (109). Ultimately what is most critical is that the tumour is appropriately visualised and targeted.

Current treatment planning for RT requires physicians to determine and contour the area to be treated based on their treatment planning computed tomography (CT) scan, which is typically fused to a magnetic resonance image (MRI). However, multimodal images are often used to accurately determine the GTV, and these can include positron emission tomography (PET) scans. In treating brain metastases, it is generally accepted that the MRI used for treatment planning should not be older than 7 to 14 days by the time of the first treatment delivery in order to reduce the risk of geographic miss, and the geometric fidelity has to be checked as that alone can lead to targeting errors (110). With technical advances in radiotherapy systems, real-time imaging is increasingly being demanded with MRI to verify the position of the target before each fraction. The MR LINAC represents a technical feat such that a MRI and a LINAC are combined, allowing for such verification and real-time on line adaptive radiotherapy (111, 112). This technology is relevant in the brain, as Tan reported on significant dynamics in the surgical cavity CTV for brain metastases treated during a 5-day course of hypofractionated stereotactic RT (113). Furthermore, there is increasing evidence of dynamic changes for high-grade glioma during a course of treatment that could otherwise result in geographic miss should the tumour not be visualised when applying small margins (114, 115).

## 5 Probabilistic factors

Due to DNA being the target of RT, the gravity of the damage incurred is associated with a level of probability. Additionally, although highly related to tumour kinetics, the stage of the cell cycle of any given cell within a tumour is another matter of probability.

### 5.1 Random gravity of DNA damage

RT is an untargeted treatment type, with no mechanism available to target the sections of DNA to be damaged. Because of this, probability dictates if sections being damaged are genes essential for cell survival or clinically irrelevant regions.

### 5.2 Cell cycle

Through differences in sister chromatid proximity and DNA damage repair pathways active at different stages in the cell cycle, discrepancies in radioresistance occur in a stage-dependent manner (10). Generally, the late S phase and G2 phase are radioresistant due to the proximity of sister chromatids during these phases, allowing for the less error-prone DNA damage repair mechanism of homologous recombination (10). Asynchronous cell cycles provide another layer of tumour heterogeneity, which can lead to additional discrepancies in RT response within the tumour (5).

## 6 Summary of radioresistance in brain metastases

Akin to how additional hallmarks of cancer had been identified since Douglas Hanahan and Robert A. Weinberg’s initial work (116), the same can be said for mechanisms of radioresistance, with many new potential mechanisms for radioresistance being identified since Peters et al.’s keynote address. Across the four categories of tumour-related factors, host-related factors, technical factors, and probabilistic factors, 19 general mechanisms for radioresistance have been identified. These include hypoxia, clonogenic/cancer stem cells, tumour kinetics, the tumour microenvironment, metabolic alterations, tumour heterogeneity, DNA damage repair, non-coding RNAs, exosomes, methylomes and autophagy as tumour-related factors; pathophysiological factors, and the volume effect and dose limiting-non-cancerous tissue as the host related factors; the machine used, technique applied and planning strategy as the technical factors; and random gravity of DNA damage and the cell cycle as probabilistic factors. Due to the broad spectrum of each mechanism, although therapeutic approaches have been identified to target many of these factors, further investigation on their application to promote radiosensitisation will be necessary. In the context of brain metastases, these general mechanisms are relevant and play potential roles in observable radioresistance. With the variety of primary cancers that may lead to brain metastases, influential differences between primary cancer types may result in significant differences in some of these mechanisms, ultimately leading to different levels of radioresistance across brain metastases between patients. With a wide variety of these mechanisms and their application to brain metastases manifesting from different primary cancer types, studies investigating these mechanisms in brain metastases with the context of their cancer type of origin would further our understanding of radioresistance in brain metastases.
